# Effect of Bariatric Surgery on Plasma Cell-Free Mitochondrial DNA, Insulin Sensitivity and Metabolic Changes in Obese Patients

**DOI:** 10.3390/biomedicines11092514

**Published:** 2023-09-12

**Authors:** Larysa V. Yuzefovych, Viktor M. Pastukh, Madhuri S. Mulekar, Kate Ledbetter, William O. Richards, Lyudmila I. Rachek

**Affiliations:** 1 Department of Pharmacology, College of Medicine, University of South Alabama, Mobile, AL 36688, USA; yuzefovych@southalabama.edu (L.V.Y.); vpastukh@southalabama.edu (V.M.P.); 2 Department of Mathematics and Statistics, College of Art and Science, University of South Alabama, Mobile, AL 36688, USA; mmulekar@southalabama.edu; 3 Department of Surgery, College of Medicine, University of South Alabama, Mobile, AL 36688, USA; kledbetter@health.southalabama.edu (K.L.); brichards@health.southalabama.edu (W.O.R.)

**Keywords:** circulating cell-free mitochondrial DNA, obesity, insulin resistance, type 2 diabetes

## Abstract

While improvement of mitochondrial function after bariatric surgery has been demonstrated, there is limited evidence about the effects of bariatric surgery on circulatory cell-free (cf) mitochondrial DNA (mtDNA) and intracellular mtDNA abundance. Plasma and peripheral blood mononuclear (PBM) cells were isolated from healthy controls (HC) and bariatric surgery patients before surgery and 2 weeks, 3 months, and 6 months after surgery. At baseline, the plasma level of short cf-mtDNA (*ND6*, ~100 bp) fragments was significantly higher in obese patients compared to HC. But there was no significant variation in mean *ND6* values post-surgery. A significant positive correlation was observed between preop plasma *ND6* levels and HgbA1c, *ND6* and HOMA-IR 2 weeks post-surgery, and mtDNA content 6 months post-surgery. Interestingly, plasma from both HC and obese groups at all time points post-surgery contains long (~8 kb) cf-mtDNA fragments, suggesting the presence of near-intact and/or whole mitochondrial genomes. No significant variation was observed in mtDNA content post-surgery compared to baseline data in both PBM and skeletal muscle samples. Overall, bariatric surgery improved insulin sensitivity and other metabolic parameters without significant changes in plasma short cf-mtDNA levels or cellular mtDNA content. Our study provides novel insights about possible molecular mechanisms underlying the metabolic effects of bariatric surgery and suggests the development of new generalized approaches to characterize cf-mtDNA.

## 1. Introduction

Obesity is associated with insulin resistance (IR), and this represents a major risk factor for metabolic syndrome (MetS), type 2 diabetes (T2D), and cardiovascular disorders such as coronary artery disease and heart failure [1,2]. It has been demonstrated that mitochondrial dysfunction contributes to obesity-related IR [3,4,5]. Previously, we and others showed that bariatric surgery rapidly improves mitochondrial respiration in morbidly obese patients [6,7]. Furthermore, even moderate weight loss and physical activity result in improvements in mitochondrial function, which results in improvements in insulin sensitivity [8]. With the increasing use of bariatric surgery for the treatment of severe obesity, it is critical to understand the molecular mechanisms responsible for the notable improvements seen after the procedure.

Overall, the publications regarding the effect of bariatric surgery and/or surgically-induced weight loss on cf-mtDNA fragments or mtDNA integrity are scarce [9,10,11,12,13]. Considering the growing importance of mtDNA analysis in obesity and IR research, the goal of this study was to evaluate plasma levels of cf-mtDNA and intracellular mtDNA content in relation to the metabolic changes before and after bariatric surgery in obese patients. Additionally, since we previously demonstrated that increased levels of plasma cf-mtDNA correlate with the degree of IR in obese T2D patients [14], in the current study we determined whether cf-mtDNA and intracellular mtDNA content vary when obese patients are grouped by presence or absence of T2D or MetS and compared to healthy controls (HC).

## 2. Materials and Methods

### 2.1. Subjects

We recruited 13 morbidly obese patients with T2D (*n* = 7), MetS (*n* = 4), and without T2D or MetS (*n* = 2) who fulfilled the National Institutes of Health criteria for bariatric surgery (body mass index (BMI) > 35 kg/m^2^). Ten patients underwent laparoscopic sleeve gastrectomy (LSG), and three patients underwent laparoscopic Roux-en-Y gastric bypass (LRYGB). Eight volunteer HC (subjects without obesity (body mass index < 30 kg/m^2^) were recruited from the general community. The presence of T2D in patients was confirmed based on a detailed clinical examination using the American Diabetes Association criteria for T2D: fasting glucose ≥126 mg/dL, HgbA1c ≥ 6.5%. The Adult Treatment Panel (ATP III) diagnostic criteria were used to establish the presence of the MetS in patients as described [15]. Post-bariatric surgery, patients were taken off medications.

All subjects were sedentary. All human studies were conducted in accordance with NIH guidelines, complied with the Declaration of Helsinki, and were approved by the Institutional Review Board of the University of South Alabama (protocol #1253267, “Effect of bariatric surgery mtDNA DAMP’s, inflammation & diabetic outcome”). All human subjects gave informed consent.

### 2.2. Metabolic Parameters and Muscle Biopsy

Each subject had a medical history, a physical examination, and blood sampling. From each bariatric surgery patient, blood was collected before surgery (BS) and 2 weeks (2 W), 3 months (3 M), and 6 months (6 M) after bariatric surgery. Metabolic parameters were measured using standard kits. Homeostasis model assessment for insulin resistance index (HOMA-IR) was calculated using the formula: HOMA-IR = [glucose (mg/dL) × insulin (µIU)/mL]/405, with fasting values. For most obese patients prior to surgery and at some points after, samples of skeletal muscle (~100 mg) were obtained from the vastus lateralis by percutaneous needle biopsy under sterile conditions with local anesthesia (lidocaine, 1%) and were snap frozen in liquid nitrogen for DNA and protein analysis.

### 2.3. Isolation of Plasma and PBM Cells

On the day of the blood sampling and/or muscle biopsy, subjects reported to the laboratory after an overnight fast (12 h). Peripheral blood (16 mL) was collected into two sterile density gradient tubes (Vacutainer with Ficoll-Hypaque solution, Becton Dickinson, Franklin Lakes, NJ, USA). Blood was fractionated by centrifugation at 1500× *g* for 30 min at 21 °C with a swinging bucket rotor, as previously described with modifications [14]. The plasma (upper) fraction was separated from PBM cells, carefully transferred into a 15 mL conical tube, and centrifuged a second time at 1800× *g* for 10 min to eliminate cell and large debris contamination. Then, it was aliquoted and stored at −80 °C until analyzed. The mononuclear-containing buffy coat fraction was suspended in 10 mL of phosphate-buffered saline (PBS; pH 7.4, Thermo Fisher, Waltham, MA, USA) and collected by centrifugation at 700× *g* for 10 min at ambient temperature as described previously [6]. This wash step was repeated once, and the mononuclear-containing buffy coat fraction was suspended in 0.1 mL of PBS (pH 7.4) and frozen at −70 °C.

### 2.4. Isolation of Plasma cf-DNA

Plasma cf-DNA was isolated using a DNeasy Blood and Tissue kit (Qiagen, Germantown, MD, USA) from aliquots of plasma samples that had been frozen at −80 °C, as we described previously with some modifications [14]. Extraction was performed with 1 mL of plasma sequentially loaded on a single column (200 μL 5 times), and the DNA was eluted with 50 μL of deionized water. The cf-DNA was stored at −20 °C for further analysis.

### 2.5. Analysis of Plasma cf-mtDNA

First, we performed qRT-PCR as we described previously, with a slight modification [16], using primers to amplify a 104 bp sequence within the mitochondrial *ND6* gene. To assess the concentration of mtDNA fragments (ng/mL) in plasma samples, we constructed a standard calibration curve using a PCR product from the whole *ND6* region using total DNA isolated from HUVEC cells. Equal amounts of eluant (5 µL) were used for qRT-PCR (SsoAdvanced™ Universal SYBR^®^ Green Supermix, #1725271, Hercules, CA, USA), according to the manufacturer’s protocol. All primers were designed by Beacon Designer 8.2 software (PREMIER Biosoft International, Palo Alto, CA, USA). Sequences of primers are provided in Supplemental Table S1. For amplification of long PCR fragments, Platinum™ SuperFi™ PCR Master (Invitrogen, Thermo Fisher Scientific, Waltham, MA, USA) was used according to the manufacturer’s recommendations, with 1 µL of DNA and two sets of overlapping mitochondrial primers, Mito 1 and Mito 2 (500 nM/each, Supplemental Table S1). The thermal cycling conditions were as follows: (1) Mito1—95 °C (2:00) + [95 °C (0:30) + 60 °C (0:30) + 68 °C (8:00)] × 30 cycles + 68 °C (5:00) + 4 °C (∞). (2) Mito2—95 °C (2:00) + [95 °C (0:30) + 55 °C (0:30) + 68 °C (8:00)] × 30 cycles + 68 °C (5:00) + 4 °C (∞). Equal amounts of PCR products were loaded in the 0.8% agarose gel with reference to the DNA standards: 1 Kb DNA ladder (Promega, Fitchburg, WI, USA) and 1 Kb DNA ladder (Invitrogen, Thermo Fisher Scientific, Waltham, MA, USA). A total of 1 ng of DNA isolated from HUVEC cells was used as a positive control, and a sample without DNA but containing water was used as a negative control. Positive and negative controls were included in each run.

### 2.6. Analysis of mtDNA Content

Total DNA was isolated from PBM cells from HC and each obese patient at baseline and 2 weeks, 3 months, and 6 months after bariatric surgery using the DNeasy Blood and Tissue Kit (Qiagen, Germantown, MD, USA). Using the same kit, we isolated total DNA from skeletal muscle samples obtained from obese patients. 20 ng of total DNA was used for qRT-PCR (Luna^®^ Universal qPCR Master Mix, #M3003L, NEB, Ipswich, MA, USA) according to the manufacturer’s protocol and PCR conditions as previously described [16]. The ratio of mtDNA to nDNA indicates the relative mtDNA content per cell in the tissue. The mtDNA/nDNA ratio was assessed by the qRT-PCR method using primers specific for the mitochondrial *ND1* gene [16] and normalizing for the amount of nDNA used in each reaction by using primers specific for *18S rRNA* [14].

### 2.7. Statistical Analyses

All data collected were analyzed using JMP Pro 16.2.0 (a product of SAS Inc., Cary, NC, USA). A significance level of 0.05 was used to determine the significance of the results. This is an unbalanced block design because of missing data for different patients at different times. Mean outcomes for four-time sets (BS, 2 W, 3 M, and 6 M) were compared using the ANOVA technique with patient as a blocking factor, i.e., measurements over different times were matched by patient. Statistically significant variation in measurements among patients and data collected for the same patients over time justifies the use of patients as a blocking factor. Post hoc comparisons for four-time sets were conducted using Tukey’s HSD. Mean outcomes for T2D-Mets groups and IR were compared using ANOVA, and post hoc comparisons with BS were conducted using Dunnett’s method. Mean outcomes for two bariatric surgery procedure groups (LSG or LRYGB) were compared using a *t*-test after accounting for differences in standard deviations, if any.

## 3. Results

### 3.1. Metabolic Parameters

Initially, we recruited 14 morbidly obese patients but excluded patient number 13 for not following instructions for postoperative blood draws. Patient number 13 ate prior to the fasting blood draws, making the outcomes of the blood tests invalid. As a result, we had 13 morbidly obese patients (9 females and 4 males) with T2D (*n* = 7), MetS (*n* = 4), and without T2D or MetS (*n* = 2), and eight HC (6 females and 2 males) subjects without obesity. The study has two groups for which separate statistical analysis was performed: (1) HC-Obese patients, BS; and (2) Obese patients at all time points. Clinical and metabolic characteristics of study groups are shown in Table 1 for Obese patients at all time points and in Supplemental Table S2 for HC and Obese BS patients.

On average, HC (47.50 ± 9.01 years) were slightly older than Obese, BS patients (41.54 ± 12.78 years), but the difference was not significant (*p* = 0.2266, Supplemental Table S2). Obese, BS patients had significantly higher BMI (*p* < 0.0001), fasting plasma glucose (*p* = 0.0102), HgbA1c (*p* = 0.0023), insulin (*p* = 0.0052), HDL (*p* = 0.0036), and triglyceride (*p* = 0.0023) levels compared to HC (Supplemental Table S2). Additionally, they were more insulin-resistant, as shown by a significantly increased HOMA-IR (*p* = 0.0237) in the Obese BS group. On average, total cholesterol and LDL for the two groups were not significantly different (Supplemental Table S2).

As can be seen by the missed data in Table 1, some parameters were not measured in the Obese group at all time points due to the missed patient visits, particularly at the time of the COVID-19 pandemic. Some parameters (HgbA1c_,_ triglycerides, HDL, and LDL) were not measured at the 2 W time point, which is indicated as *n* = 0. Mean outcomes for clinical and metabolic data over four time points (BS, 2 W, 3 M, and 6 M) were compared using the ANOVA technique with patients as a blocking factor, i.e., measurements over different times were matched by patient. Significant patient-to-patient variation was observed in the following metabolic parameters, indicating blocking (or matching) by patient was justified (Table 1): BMI (*p* < 0.0001), glucose (*p* < 0.0001), HgbA1c (*p* < 0.0023), triglycerides (*p* = 0.0002), total cholesterol (*p* = 0.0213), HDL (*p* = 0.0002), and plasma *ND6* (*p* = 0.0443).

Bariatric surgery, on average, reduced BMI over time (Table 1). Mean BMI differed significantly at least two different times (*p* < 0.0001, power = 1.0). In fact, the mean BMI decreased as time advanced. Post hoc analysis using Tukey’s test shows that mean BMI dropped significantly from BS (49.90) to 2 W (44.49) and then to 3 M (39.54). Although, the 6 M (36.61) mean BMI was lower than that for 3 M (39.54), the difference was not statistically significant, but the 6 M BMI was significantly different from the BS and 2 W BMIs. Although the mean glucose level decreased steadily from 125.31 at BS to 85.30 at 6 M, the difference was not statistically significant (*p* = 0.1317). Mean HgbA1c differed significantly for at least two different times (*p* = 0.0151, power = 0.99); in fact, it decreased over time. Post hoc analysis using Tukey’s test showed a significant drop from BS (7.04) to 3 M (5.39) and 6 M (5.30), but a decrease from 3 M (5.39) to 6 M (5.30) was not statistically significant. Insulin levels did not differ significantly from patient to patient (*p* = 0.3424); however, the mean insulin level differed significantly for at least two different times (*p* = 0.0034, power = 0.751). In fact, it decreased over time from BS to 3 M (BS: 50.29, 2 W: 16.35, and 3 M: 10.58) only to increase to 14.40 at 6 M. Post hoc analysis using Tukey’s test showed a significant decrease in mean insulin level from BS to 2 W, 3 M, and 6 M; however, the decrease from 2 W to 3 M was not significant, nor was the increase from 3 M to 6 M. HOMA-IR levels did not differ significantly from patient to patient (*p* = 0.0780), but mean the HOMA-IR level differed significantly at least two different times (*p* = 0.0126, power = 0.77). In fact, it decreased from BS (18.66) to 2 W (4.52). The drop from BS to 3 M (2.40) was also significant. But it then increased to 6 M (3.58), though not significantly. Post hoc analysis using Tukey’s test showed a significant decrease in mean HOMA-IR level from BS to 2 W; however, later changes in HOMA-IR level over time were not statistically significant. HOMA-IR levels among patients at BS varied the most, decreasing with time only to increase at 6 M.

Triglyceride levels varied significantly from patient to patient (*p* = 0.0002). Mean triglyceride levels differed significantly at least two different times (*p* = 0.0026, power = 1.0). In fact, mean triglyceride levels decreased as time advanced. Post hoc analysis using Tukey’s test shows that mean triglyceride levels at both 3 M (99.50) and 6 M (91.57) were significantly lower than BS (159.85); however, the decrease from 3 M to 6 M was not statistically significant. Total cholesterol levels differed significantly by patient (*p* = 0.0213), but no significant differences in mean cholesterol levels were observed over time (*p* = 0.9287, BS: 167.23, 3 M: 168.13, and 6 M: 169.00). Significant variation in HDL levels was observed among patients (*p* = 0.0002). Mean HDL levels differed significantly for at least two time periods (*p* = 0.0113, power = 1.0). A steady increase in HDL levels with time was observed. Post hoc analysis using Tukey’s test indicates a slight but not statistically significant increase in mean HDL level from BS (40.08) to 3 M (43.88). The increase in mean HDL level from BS to 6 M (51.57) as well as from 3 M to 6 M is significant. No significant variation in LDL levels was observed among patients (*p* = 0.0773) or at three different times (*p* = 0.8800).

Comparison of clinical and metabolic parameters among types of bariatric surgery (LSG or LRYGB groups) showed that the 3 M mean insulin level was significantly lower among LRYGB patients than LSG patients (5.80 vs. 11.77, *p* = 0.0040, Supplemental Table S3). Also, the 3 M mean HOMA-IR level was significantly lower among LRYGB patients than LSG patients (1.18 vs. 2.71, *p* = 0.0066, Supplemental Table S3).

### 3.2. Effect of Obesity and Bariatric Surgery on Plasma cf-mtDNA

**Figure 1:**
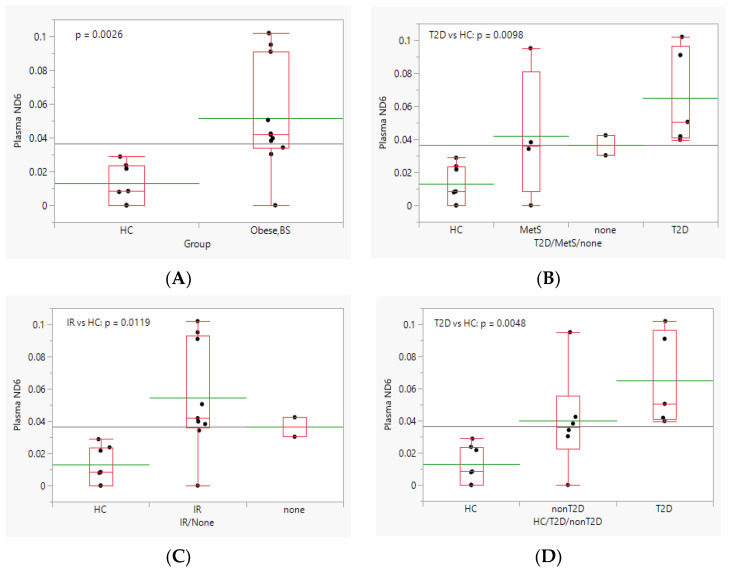
Effect of (**A**) obesity, (**B**) MetS, (**C**) IR, and (**D**) T2D on plasma *ND6* level. The data are presented as boxplots, with the mean marked by green lines.

First, we analyzed short cf-mtDNA fragments (104 bp) within the mitochondrial *ND6* gene using qRT-PCR. Analysis of *ND6* cf-mtDNA in plasma was performed separately for two groups: (1) HC-Obese, BS group, and (2) Obese group, at all times (BS, 2 W, 3 M, and 6 M). Upon comparing the level of short cf-mtDNA in plasma from HC and Obese BS patients, we found that the level of plasma cf-mtDNA *ND6* fragments was five times greater in Obese BS (0.05 ± 0.03) compared to HC (0.01 ± 0.01, *p* = 0.0026, Figure 1A). Additionally, when patients in the Obese BS group were classified as MetS, T2D, or none, we found that mean plasma *ND6* levels were significantly higher in samples of T2D than HC (Dunnett’s method for comparison with HC, *p* = 0.0098, Figure 1B, Supplemental Table S4). Similarly, a significantly higher mean plasma *ND6* level was observed in the IR in Obese BS patients compared to HC (Dunnett’s method for comparison with HC, *p* = 0.0119, Figure 1C, Supplemental Table S5).

Comparison of plasma *ND6* levels among HC/T2D/nonT2D groups resulted in significant differences in the mean levels of at least two groups (Figure 1D, ANOVA, *p* = 0.0085). Post hoc analysis using Dunnett’s method showed that mean plasma *ND6* levels of T2D patients were significantly higher than HC (Figure 1D, *p* = 0.0048); however, those of non-T2D patients were not significantly different than HC (Figure 1D, *p* = 0.1177).

**Figure 2:**
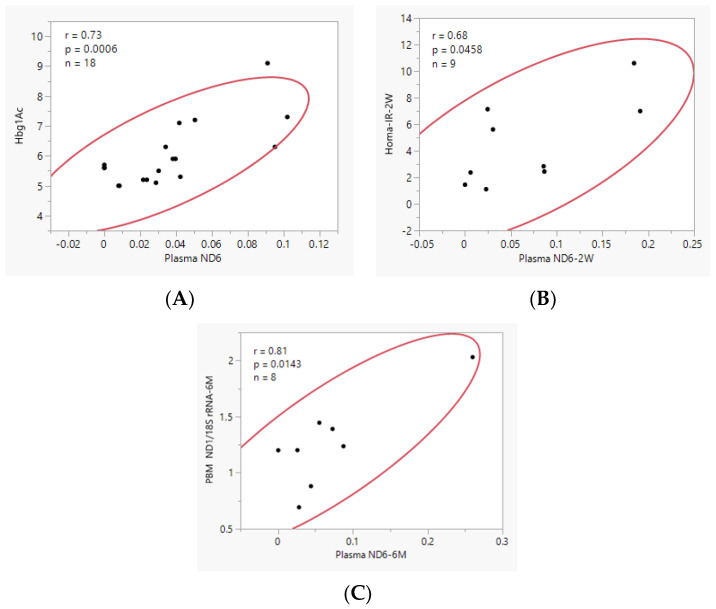
A significant positive correlation as measured by Pearson’s correlation coefficient was observed between plasma levels of *ND6* and (**A**) pre-surgery HgbA1c in the HC-Obese, BS group of patients (*n* = 18); (**B**) HOMA-IR measured 2 W post-surgery (*n* = 9); and (**C**) PBMN mtDNA content measured 6 M post-surgery (*n* = 8). Red ellipse is the 95% bivariate normal ellipse.

A significant positive correlation was observed between HgbA1c and plasma *ND6* (r = 0.73, *n* = 18, *p* = 0.0006, Figure 2A). However, when HC and obese patient data were separated, a negative correlation was observed for HC, which was not statistically significant probably due to the small sample size (r = −0.55, *n* = 7, *p* = 0.1967, Supplemental Figure S1), and a significant positive correlation was observed for Obese BS patients (r = 0.63, *n* = 11, *p* = 0.0362, Supplemental Figure S1).

Post-surgery analysis of plasma *ND6* levels in obese patients showed that ND6 values varied significantly among patients (*p* = 0.0443, Table 1). Although mean plasma cf-mtDNA *ND6* levels at three post-operative points were slightly higher compared to baseline, the differences were not significant (*p* = 0.8539, Supplemental Figure S2). Interestingly, comparison of the Obese MetS, T2D, and without MetS (designated as “none”) groups showed that mean plasma *ND6* levels were higher in T2D patients’ samples at all time points except at 3 M after bariatric surgery compared to those in the other two groups (Supplemental Table S6).

A significant positive correlation was observed between plasma levels of plasma *ND6* and HOMA-IR, 2 W post-surgery (r = 0.68, *p* = 0.0458, Figure 2B), and PBMN *ND1*/*18S rRNA*, 6 M post-surgery (r = 0.81, *p* = 0.0143, Figure 2C). No significant correlation was observed between BMI and plasma *ND6* for any of the 4 time periods.

**Figure 3:**
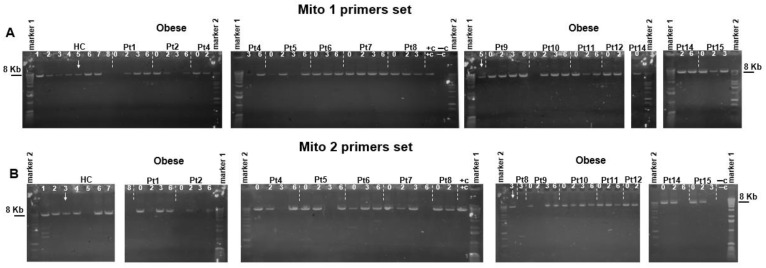
Representative gel electrophoresis of long-range PCR analysis. Using total DNA isolated from plasma as a template showed that plasma from HC and obese patients (all time points) contained (**A**) Mito1 (~8.4 kb) or (**B**) Mito2 (~8 kb) long overlapping amplicons that covered the full length mitochondrial genome. 1 ng of total DNA isolated from HUVEC cells was used as a positive control (+c), and a sample without DNA but containing water was used as a negative control (−c). No signal was detected while using the negative control. To compare the level of amplicons between obese and HC, a PCR sample from HC #5 was loaded on every gel analysis for long-range PCR with the Mito1 set of primers (indicated with an arrow above the gel). A sample from HC #3 was also loaded in every gel for comparison while using the Mito2 primer set, which is indicated with an arrow above the gel. Marker 1-1 Kb DNA ladder (Invitrogen) and Marker 2-1 Kb DNA ladder (Promega).

Second, we analyzed the presence of long cf-mtDNA fragments using two pairs of overlapping mitochondrial sequences covering the whole mtDNA genome for the identification of long mtDNA fragments. The location of sequences and size of the amplified fragments are shown on the scheme presented in Supplemental Figure S3. Long-range PCR analysis of samples from the Obese group at all time points after BS revealed that plasma from most samples contains long amplicons (~8 kb), suggesting the presence of near intact and/or whole mitochondrial genomes (Figure 3A,B). Although the majority of HC samples also showed long amplicons in their plasma, in some HC (#8 for Mito1 and #5 and 8 for Mito2), bands for long amplicons were not even observed, suggesting either the absence or a very low quantity of the long fragments in their pool of plasma long cf-mtDNA (Figure 3A,B). Although the inferences about differences between controls and obese samples should be qualified by the limitation that the data for long PCR products is not quantitative, the abundance of long PCR products was noticeably greater in samples from obese patients (Figure 3A,B) compared to samples from the HC group, at least for the Mito1 primers dataset. Therefore, not only were the short *ND6* fragments accumulated in plasma from obese patients, but also the complete mitochondrial genome, as determined by long-range PCR amplifications of 2 amplicons (~8 kb each, Figure 3A,B).

### 3.3. Effect of Obesity and Bariatric Surgery on mtDNA Content in PBM Cells and Skeletal Muscle

Next, we evaluated relative mtDNA content (mtDNA/nDNA ratio) using total DNA isolated from PBM or skeletal muscle. First, we used total DNA isolated from PBM cells from HC and each obese patient at baseline and at the time points indicated above after bariatric surgery. Although not statistically significant, the *ND1*/*18S rRNA* ratio in PBM cells on average was observed to be higher among Obese BS patients (1.0 ± 0.31) compared to HC (0.76 ± 0.32) (*p* = 0.1013, Supplemental Figure S4A). When patients in the Obese BS group were further classified as MetS (*n* = 4), T2D (*n* = 7), and none (*n* = 2), they all had a higher mean *ND1*/*18S rRNA* ratio in PBM cells than HC, but the differences were not statistically significant, probably due to small sample sizes (Supplemental Figure S4B, Supplemental Table S4). Interestingly, when Obese BS patients were classified as IR vs. none, their mean PBM cell *ND1*/*18S rRNA* ratio, although not significant, was higher compared to HC, with the “none” group having the highest mean (*p* = 0.0976, ANOVA, Supplemental Figure S4C, Supplemental Table S5). It is important to note that the “none” group had only two patients, and the IR group had two outliers on the higher end. Comparison of mean PBM cell *ND1*/*18S rRNA* levels by HC/T2D/non T2D groups resulted in no significant differences (*p* = 0.2021, ANOVA, Supplemental Figure S4D). Also, note an outlier in the T2D group (Supplemental Figure S4D). The mean PBM cell *ND1*/*18S rRNA* level was observed to be higher in obese non-T2D patients compared to HC, but the difference was not significant (Supplemental Figure S4D).

**Figure 4:**
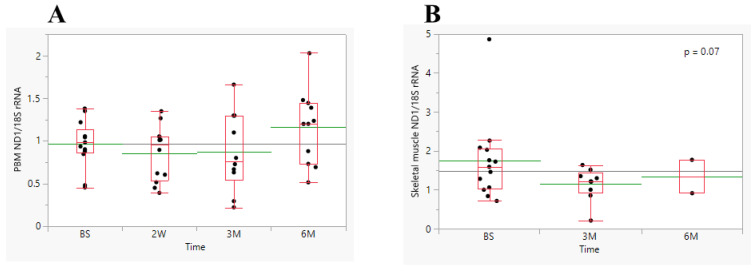
Effect of bariatric surgery on mtDNA abundance. (**A**)—mtDNA content in PBM prior to and follow-up post-surgery (*n* = 10–13) and (**B**)—mtDNA content in skeletal muscle prior to surgery and follow-up post-surgery (*n* = 2–13). The data are presented as boxplots, with the mean marked by green lines.

Comparison of PBM *ND1*/*18S rRNA* levels in obese patients at BS and three different post-surgery times showed no significant variation among patients (*p* = 0.3311) or over the different times it was measured (*p* = 0.3759, Table 1 and Figure 4A). Additionally, we analyzed total DNA isolated from skeletal muscle samples obtained from some obese patients at various time points post-surgery. As shown in Figure 4B and Table 1, no significant variation in the mean skeletal muscle *ND1*/*18S rRNA* ratio was observed among patients (*p* = 0.3104) or among three different times (*p* = 0.07). Additionally, the 2 W mean PBM *ND1*/*18S rRNA* level was significantly lower among LRYGB patients than LSG patients (0.52 vs. 0.96, *p* = 0.0025, Supplemental Table S3).

## 4. Discussion

Our study demonstrated that while bariatric surgery reduced BMI, it did not significantly change plasma short cf-mtDNA levels or mtDNA content in PBM cells or skeletal muscle. Surprisingly, we found that plasma from both HC and obese patients contained long (~8 kb) cf-mtDNA fragments, which cover near intact and/or whole mitochondrial genomes.

Metabolic improvement after bariatric surgery going beyond mere weight loss, and given the increasing use of bariatric surgery, it is critical to understand the physiologic basis of its metabolic benefits, including resolution of T2D. Bariatric surgery has variable effects on mitochondria, reviewed in [17], including improvement in mitochondrial respiration in PBM cells [6], liver [13], and skeletal muscle [6,7]. Additionally, bariatric surgery induced alterations in skeletal muscle expression of genes involved in calcium/lipid metabolism and mitochondrial function, which were associated with subsequent distinct methylation patterns at 52 weeks after surgery [18]. Furthermore, a recent study demonstrated that laparoscopic Roux-en-Y gastric bypass augmented autophagy/mitophagy markers and decreased mitochondrial membrane potential in leukocytes, improving mitochondrial turnover as a consequence [19].

Despite the intensive studies on mitochondrial physiology, only a few studies have examined circulatory or urine cf-mtDNA [9,10,11] and mtDNA/nDNA content [12,13] in obese patients after bariatric surgery. Previously, it has been shown that bariatric surgery after 6 months reduced urinary mt*ND1* and mt*COX3* copy numbers, as well as serum mt*COX3* copy numbers, only in patients with obesity with T2D [10]. Additionally, the same group demonstrated that bariatric surgery reduces the elevated urinary mtND1 copy number in obese patients with an initial high baseline mt*ND1* copy number 6 months after surgery [10]. Also, a study in obese Asian Indian patients with T2D demonstrated a significant reduction in circulatory mtDNA copy number at 6 and 12 months post-bariatric surgery [11]. In contrast with these previous reports, we did not observe a reduced level of short cf-mtDNA post-surgery. Simultaneously, we observed a significant increase in short plasma *ND6* cf-mtDNA in obese patients compared to HC (Figure 1A). Additionally, we found that the mean plasma *ND6* level was significantly higher in samples of T2D than HC (Figure 1B), which is consistent with our [14] and others previous reports for an increase in cf-mtDNA in patients with T2D [20]. Similarly, we observed a significantly higher level of plasma short cf-mtDNA in the IR in Obese BS compared to HC (Figure 1C). These findings are in line with a recent study of healthy adolescents that showed that circulating cf-DNA is associated with the risk of metabolic syndrome, not obesity per se [21]. Furthermore, this study found an association between cf-mtDNA and one of the markers of oxidative stress, advanced oxidation protein products [21]. Recently, the use of circulatory cf-DNA and cf-mtDNA has received a lot of attention due to their attractive diagnostic, prognostic, and risk assessment potential. In light of this, blood levels of cf-DNA have been found to be correlated with IR and other obesity-related cardiometabolic risk factors in clinically healthy individuals, leading to a proposal to use cf-DNA as an auxiliary risk marker for cardiometabolic disease [22]. Nevertheless, studies to determine cf-mtDNA as a potential biomarker for predicting the outcome of bariatric surgery are in the preliminary stages.

Analysis of plasma *ND6* cf-mtDNA in obese patients after surgery did not show significant changes over the time after surgery (Supplemental Figure S2), which was different from the previous reports indicating a decrease in cf-mtDNA copy number post-surgery [9,10,11]. The discrepancy between our results and results from previous studies can be influenced by several potential factors, including source (serum, plasma, or urine) and approaches for cf-DNA isolation, analysis, and interpretation of results. As opposed to our study, in which we performed absolute quantification of cf-mtDNA in plasma, in all previous related studies, the authors measured the relative cf-mtDNA amount, which was calculated as a ratio of cf-mtDNA/cf-nDNA in either serum or urine. Because of the relative nature of the analysis, we believe that the above-mentioned previous studies do not support the selective release/accumulation of cf-mtDNA associated with obesity or T2D and its selective decrease with weight loss after bariatric surgery. Additionally, we believe that the initial and especially post-surgery BMI can be a contributing factor to the discrepancy in the analysis as well. Also, it needs to be mentioned that even after profound weight loss our patients were still severely obese, with a BMI ~36.6 at 6 M post-surgery. We need to point out that both pre- and post-operative BMI for our patients were much greater than in related publications [9,10,11], which most likely contributed to the outcomes of our study. It must also be highlighted that gender differences could affect our findings. Indeed, the above-mentioned related studies have had a higher male percentage: ~41% (9) and ~37% [10,11] vs. ~31% male in our study. Interestingly, similar to our study, the decrease in body weight and fat percentage did not result in changes in cf-DNA levels six months after bariatric surgery [23]. Additionally, we need to mention that some data for 3 M and 6 M post-surgery were missing for different patients at different times, partially due to the COVID-19 pandemic-related missed visits (Table 1). This may also affect the outcomes for the dynamic for plasma *ND6* level. Regarding the association between cf-mtDNA level and markers of T2D in HC and Obese patients, we observed a significant positive correlation between HgbA1c and plasma *ND6* (Figure 2A). However, when HC and obese patient data were separated, no correlation between *ND6* and HgbA1c was observed for HC (Supplemental Figure S1), but we note that 100% of the HC had a normal HgbA1c and *ND6* level ≤ 0.3 ng/mL, and all of the obese patients at baseline had a *ND6* ≥ 0.4 ng/mL (Figure 2A). This suggests that plasma levels of *ND6* above 0.4 ng/mL are pathologic and associated with increasing levels of HgbA1c.

Analysis of long-range PCR showed that plasma from HC and Obese patients (at all time points after bariatric surgery) contained long overlapping amplicons that covered the full-length mitochondrial genome (Figure 3A,B). Therefore, not only were the short *ND6* fragments accumulated in plasma from obese patients but also the complete mitochondrial genome. The latter may be an adaptive mechanism to preserve mitochondrial function and bioenergetics (i.e., via restoration of OXPHOS), since cells may share/transfer whole healthy mitochondria and/or mtDNA to each other when they recover from the stress of obesity and diabetes. Previous studies showed that isolated mitochondria can be transferred to any cell type via simple coincubation or brief centrifugation in vitro [24,25], reviewed in [26]. Furthermore, the injection of autologous or non-autologous mitochondria has been effective in treating injury and various diseases [27,28], reviewed in [29]. In line with this, a recent paper has shown that exogenous healthy mitochondria are preferentially trafficked to cells and tissues in which mitochondria are damaged [30], which has implications for the delivery of therapeutic agents to injured or diseased sites. Whether this process happened after bariatric surgery needs further investigation. As we mentioned above, even after 6 M post-surgery, patients were still morbidly obese, although their metabolic parameters, such as HOMA-IR and HgbA1c, improved significantly. While it needs to be interpreted with limitations noted in the Results part, we can speculate that the observed increase in long cf-mtDNA may represent an adaptive response in order to compensate for the cells’ bioenergetic needs that ultimately provide improvement in metabolic parameters and IR. The main unanswered scientific question here is: does bariatric surgery stimulate adaptive responses through mitochondria and/or mtDNA transfer? In this context, most likely, the possibility of using quantitative analysis of cf-mtDNA as an indicator of bariatric surgery results prognostic needs further investigation, with greater sample size and improvement and standardization of methods of detection.

To the best of our knowledge, this is the first report in which the accumulation of long plasma mtDNA fragments was analyzed in obesity and bariatric surgery research. Numerous previous studies have demonstrated mitochondrial transfer [24,25], reviewed in [26], between mammalian cells in culture and in vivo in its application as a therapeutic tool for treating injury and various diseases [27,28], reviewed in [29]. The study by Dache et al. reported that blood preparation with resting platelets contains respiratory-competent cell-free mitochondria in their normal physiological state [31]. Additionally, the authors showed that normal and tumor-cultured cells are able to secrete their mitochondria [31]. Furthermore, a few studies demonstrated a horizontal transfer of mtDNA [32,33]. Sansone et al. demonstrated that the horizontal transfer of mtDNA from extracellular vesicles acts as an oncogenic signal, promoting an exit from dormancy in hormonal therapy-resistant breast cancer [32]. A more recent study demonstrated that chimeras composed of cells with wild-type and mutant mtDNA exhibited increased trafficking of wild-type mtDNA to mutant cells, suggesting that horizontal mtDNA transfer may be a compensatory mechanism to restore compromised mitochondrial function [33]. A recent review discussed that circulating cf-mtDNA in its physiological forms in human blood is unlikely to be pro-inflammatory [34], and its relevance and physiological role remain to be established. Likewise, the origin and specificity of our findings of plasma long cf-mtDNA fragments for bariatric surgery-related improvement in metabolic parameters and IR are unknown and will require further testing.

Interestingly, mtDNA content tended to be slightly increased in PBM cells (Figure 4A), but decreased in skeletal muscle at 6 M after surgery compared to BS (Figure 4B), suggesting a tissue-specific effect of bariatric surgery on mtDNA content. Our finding is in line with a recent study showing that obesity has an opposing association with mitochondrial respiration in adipose and liver tissue with no overall association with NAFLD severity; however, bariatric surgery increased hepatic mtDNA/nDNA content 12 months after bariatric surgery, which appeared to be driven primarily by a substantial increase in mitochondrial biogenesis [13]. Additionally, it was reported that the copy number of mtDNA in various fat stores was higher in obese patients with T2D than in obese patients without diabetes or in control subjects [35]. This finding [35] was different from our study since mtDNA content in PBM cells was slightly increased in non-diabetics compared to both HC and T2D patients, although the difference was not significant (Supplemental Figure S4D). When compared to HC, mtDNA content in PBM cells was also slightly greater, regardless of the patient’s T2D or MetS status (Supplemental Figure S4B). A significant positive correlation was observed between plasma levels of plasma *ND6* and mtDNA content in PBM cells 6 M post-surgery (Figure 2C).

A limitation of this study was the relatively small sample size, the missing data for different patients at different times, and the short time of follow-up post-surgery. Future studies with a larger sample size and designed to address limits and methodological issues related to the isolation and analysis of cf-mtDNA are needed to generalize these results. Regarding technical limitations, we need to highlight that the current methods of cf-mtDNA quantitation in plasma are time-consuming, lack reproducibility, and do not indicate selective release and/or accumulation of cf-mtDNA in plasma. Variations in DNA isolation methods, normalization of cf-mtDNA to cf-nDNA, or generation of a standard curve altogether limit the reproducibility or clinical utility of mtDNA analysis in previous reports. In this context, a recent report using droplet digital PCR provides absolute DNA quantification without the need for a standard curve. Importantly, this method is more sensitive than conventional qRT-PCR at low concentrations of cf-mtDNA, making it feasible to analyze DNA in plasma directly without a DNA isolation step [36].

In conclusion, we found that bariatric surgery decreased weight and improved insulin sensitivity and other metabolic parameters in obese patients without significant changes in plasma short cf-mtDNA levels or cellular mtDNA content. However, a significant correlation between preop *ND6* levels and HgbA1c and *ND6* levels and insulin resistance (HOMA-IR) at 2 weeks post-op suggests that one of the potential drivers of insulin resistance may be short cf-mtDNA fragments such as *ND6.* These findings, along with the presence of long cf-mtDNA fragments, may underlie the clinical importance of cf-mtDNA as a mechanism explaining the effects of bariatric surgery on the improvement of insulin sensitivity. Further studies need to test the structure, potential characteristics, determinants, and origin of cf-mtDNA associated with metabolic improvements following surgery.

## Figures and Tables

**Table 1:** Summary of clinical, metabolic, plasma cf-mtDNA data, and mtDNA content of obese bariatric surgery patients before surgery and at all time points post-surgery.

Obese Only	Time	*n*	Mean	Std Dev	R^2^	*p* (Patient)	*p* (Time)			
BMI	BS	13	49.90	7.91	0.88	<0.0001	<0.0001	A		
	2 W	12	44.49	7.59					B	
	3 M	10	39.54	6.99						C
	6 M	10	36.61	6.62						C
Glucose	BS	13	125.31	43.64	0.78	<0.0001	0.1317			
	2 W	12	117.17	72.08						
	3 M	10	89.30	9.68						
	6 M	10	85.30	20.24						
HgbA1c	BS	13	7.04	1.73	0.89	0.0023	0.0151	A		
	2 W	0								
	3 M	8	5.39	0.31					B	
	6 M	7	5.30	0.29					B	
Insulin	BS	12	50.29	42.57	0.56	0.3424	0.0034	A		
	2 W	12	16.35	10.14					B	
	3 M	10	10.58	4.24					B	
	6 M	10	14.40	14.90					B	
HOMA-IR	BS	12	18.66	22.50	0.59	0.0780	0.0126	A		
	2 W	12	4.52	3.07					B	
	3 M	10	2.40	1.16					B	
	6 M	10	3.58	5.01				A	B	
Triglycerides	BS	13	159.85	77.95	0.92	0.0002	0.0026	A		
	2 W	0								
	3 M	8	99.50	13.77					B	
	6 M	7	91.57	31.97					B	
Total Cholesterol	BS	13	167.23	31.93	0.75	0.0213	0.9287			
	2 W	0								
	3 M	8	168.13	27.53						
	6 M	7	169.00	28.61						
HDL	BS	13	40.08	6.91	0.91	0.0002	0.0113		B	
	2 W	0								
	3 M	8	43.88	11.70					B	
	6 M	7	51.57	14.59				A		
LDL	BS	13	95.08	29.76	0.69	0.0773	0.8800			
	2 W	0								
	3 M	8	104.44	20.80						
	6 M	7	99.14	12.62						
Plasma *ND6*	BS	6	0.06	0.03	0.66	0.0443	0.8539			
	2 W	9	0.07	0.07						
	3 M	7	0.08	0.06						
	6 M	8	0.07	0.08						
Skeletal muscle *ND1/18SrRNA*	BS	13	1.74	1.06	0.69	0.3104	0.0700			
	2 W	0								
	3 M	9	1.14	0.42						
	6 M	2	1.34	0.61						
PBM *ND1*/*18S rRNA*	BS	13	0.97	0.28	0.39	0.3311	0.3759			
	2 W	12	0.85	0.32						
	3 M	10	0.87	0.46						
	6 M	11	1.16	0.44						

BS—before surgery. The *n* is variable because of missing data for different patients at different times. Levels not connected by the same letter are significantly different.

## Data Availability

All relevant data are within the manuscript and its Supplementary Files.

## Supplementary Materials

The following supporting information can be downloaded at: https://www.mdpi.com/article/10.3390/biomedicines11092514/s1, Figure S1: Correlation between HgbA1c and plasma *ND6* levels prior to surgery with patients separated as HC and Obese, BS (n = 7, HC; n = 11, Obese, BS); Figure S2: Effect of bariatric surgery on plasma cf-mtDNA. *ND6* level in obese patients prior to surgery and at the indicated time points follow up post-surgery. Data are presented as boxplots with mean marked by green lines, n = 6–9; Figure S3: Schematic of location of primers and long overlapping mtDNA sequences (Mito1 and Mito2) amplified whole mitochondrial genome during long-range PCR; Figure S4: Effect of obesity, MetS and T2D on the mtDNA abundance in PBM cells. Data are presented as boxplots with mean marked by green lines. (**A**) mtDNA content in PBM cells from Obese patients and HC (n = 8–13). (**B**–**D**) mtDNA abundance in PBM cells demonstrated by group (**B**) data in Obese, BS group were plotted according to the MetS, T2D or none (n = 2–8); (**C**) data in Obese, BS group were plotted according to the IR or none (no IR); n = 2–8 subjects per group; (**D**) data in Obese, BS group were plotted according to the T2D or non-T2D (n = 6–8); Table S1: Primers for mtDNA and nDNA; Table S2: Summary of clinical, metabolic, cf-mtDNA data, and mtDNA content of patients, HC-Obese, BS group; Table S3: Comparison of clinical and metabolic parameters, plasma cf-mtDNA data, and mtDNA content among type of bariatric surgery; Table S4: Comparison of *ND6* and PBM *ND1/18SrRNA* in HC and Obese, BS by factor: MetS, T2D or none; Table S5: Comparison of *ND6* and PBM *ND1/18SrRNA* in HC and Obese, BS group where Obese group is classified as IR or none; Table S6: Comparison of plasma *ND6* in Obese group by MetS, T2D, or none.

## Abbreviations

BMI, body mass index; cf, cell-free; BS, before surgery; HC, healthy controls; HDL, high-density lipoprotein; HOMA-IR, homeostasis model assessment for insulin resistance; LDL, low-density lipoprotein; LSG, laparoscopic sleeve gastrectomy; LRYGB, laparoscopic Roux-en-Y gastric bypass; MetS, metabolic syndrome; mtDNA, mitochondrial DNA; n-number of patients; nDNA, nuclear DNA; PBM, peripheral blood mononuclear; PBS, phosphate- buffered saline; Pt, patients; T2D, type 2 diabetes.
