# Supplementary material for: Effect of Bariatric Surgery on Plasma Cell-Free Mitochondrial DNA, Insulin Sensitivity and Metabolic Changes in Obese Patients
Source: Biomedicines. 2023 Sep 12;11(9):2514. doi: 10.3390/biomedicines11092514 (PMC10526219; doi:10.3390/biomedicines11092514)
Supplement: Supplementary file 1 [file biomedicines-11-02514-s001.zip › biomedicines-2521664-supplementary.pdf]

Supplemental Information.

Supplemental Table S1. Primers for mtDNA and nDNA.

Gene	Forward	Reverse
Short mt-*ND6*	5'- CCATCGCTGTAGTATATCCAA-3'	5'-TCGGGTGTGTTATTATTCTGA-3'
Long Mito1	5'- AAATCTTACCCCGCCTGTTT -3'	5'- AATTAGGCTGTGGGTGGTTG-3'
Long Mito2	5'- GCCATACTAGTCTTTGCCGC -3'	5'- GGCAGGTCAATTTCACTGGT-3'

Supplemental Table S2. Summary of clinical, metabolic, cf-mtDNA data, and mtDNA content of patients, HC-Obese, BS group

Parameter	Group	N	Mean	Std Dev	P (t test)
Age (years)	HC	8	47.50	9.01	0.2266
	Obese,BS	13	41.54	12.78	
BMI	HC	8	25.18	2.22	<0.0001
	Obese,BS	13	49.90	7.91	
Glucose	HC	8	87.88	10.08	0.0102
	Obese,BS	13	125.31	43.64	
HgbA1c	HC	8	5.19	0.29	0.0023
	Obese,BS	13	7.04	1.73	
Insulin	HC	8	7.50	6.16	0.0052
	Obese,BS	12	50.29	42.57	
Homa-IR	HC	8	1.61	1.24	0.0237
	Obese,BS	12	18.66	22.50	
Triglycerides	HC	8	74.25	27.38	0.0023
	Obese,BS	13	159.85	77.95	
Total Cholesterol	HC	8	188.50	32.45	0.1632
	Obese,BS	13	167.23	31.93	
HDL	HC	8	66.75	17.98	0.0036
	Obese,BS	13	40.08	6.91	
LDL	HC	8	107.00	30.20	0.3912
	Obese,BS	13	95.08	29.76	
Plasma *ND6*	HC	7	0.01	0.01	0.0026
	Obese,BS	11	0.05	0.03	
PBMN *ND1/18S rRNA*	HC	8	0.76	0.32	0.1013
	Obese,BS	13	1.00	0.31	

The data for insulin and HOMA-IR were missing for Obese Pt4.

Supplemental Table S3. Comparison of clinical and metabolic parameters, plasma cf-mtDNA data, and mtDNA content among type of bariatric surgery

		Bariatric surgery procedure, LRYGB/LSG						p
		LRYGB			LSG			(t- test)
	**Time**	**N**	**Mean**	**Std Dev**	**N**	**Mean**	**Std Dev**	
BMI	BS	3	50.07	7.56	10	49.85	8.41	0.9684
	2W	3	46.13	8.39	9	43.94	7.76	0.7151
	3M	2	40.30	8.06	8	39.35	7.31	0.8978
	6M	2	34.00	6.51	8	37.26	6.92	0.6075
Glucose	BS	3	151.33	70.50	10	117.50	33.78	0.4966
	2W	3	171.33	141.05	9	99.11	26.50	0.4691
	3M	2	82.00	4.24	8	91.13	9.95	0.1126
	6M	2	69.00	9.90	8	89.38	20.44	0.1191
HgbA1c	BS	3	7.83	3.01	10	6.80	1.30	0.6159
	2W	0			0			
	3M	1	5.10		7	5.43	0.31	
	6M	1	5.00		6	5.35	0.28	
Insulin	BS	3	66.17	72.12	9	45.00	32.65	0.6662
	2W	3	10.07	6.91	9	18.44	10.47	0.1700
	3M	2	5.80	0.85	8	11.77	3.86	**0.0040**
	6M	2	5.40	2.12	8	16.65	16.00	0.0920
HOMA-IR	BS	3	31.65	43.42	9	14.33	11.84	0.5626
	2W	3	3.68	2.32	9	4.80	3.36	0.5476
	3M	2	1.18	0.23	8	2.71	1.09	**0.0066**
	6M	2	0.95	0.49	8	4.24	5.45	0.1338
Triglycerides	BS	3	147.33	38.55	10	163.60	87.77	0.6590
	2W	0			0			
	3M	1	107.00		7	98.43	14.51	
	6M	1	104.00		6	89.50	34.51	
Total Cholesterol	BS	3	142.67	56.04	10	174.60	20.00	0.4285
	2W	0			0			
	3M	1	180.00		7	166.43	29.29	
	6M	1	164.00		6	169.83	31.25	
HDL	BS	3	36.67	5.03	10	41.10	7.28	0.2867
	2W	0			0			
	3M	1	33.00		7	45.43	11.72	
	6M	1	35.00		6	54.33	13.84	
LDL	BS	3	76.33	50.58	10	100.70	21.46	0.4943
	2W	0			0			
	3M	1	126.00		7	101.36	20.40	
	6M	1	108.00		6	97.67	13.14	
Plasma *ND6*	BS	2	0.05	0.04	4	0.06	0.03	0.7889
	2W	2	0.03	0.01	7	0.08	0.08	0.1143
	3M	2	0.14	0.06	5	0.05	0.05	0.2050
	6M	1	0.04		7	0.08	0.09	
Skeletal muscle *ND1/18S rRNA*	BS	3	1.05	0.47	10	1.95	1.11	0.0734
	2W	0			0			
	3M	2	1.03	0.25	7	1.17	0.47	0.6055
	6M	0			2	1.34	0.61	
PBM *ND1/18S rRNA*	BS	3	0.91	0.03	10	0.99	0.32	0.4482

		Bariatric surgery procedure, LRYGB/LSG						p
		LRYGB			LSG			(t- test)
	Time	N	Mean	Std Dev	N	Mean	Std Dev	
	2W	3	0.52	0.08	9	0.96	0.30	**0.0025**
	3M	2	0.80	0.71	8	0.89	0.45	0.8852
	6M	2	1.18	0.42	9	1.16	0.46	0.9588

Note three comparisons which showed significant difference in mean outcome:

1. Three-month mean insulin level is significantly lower among LRYGB patients than LSG patients (5.80 vs 11.77, p = 0.0040).
2. Three-month mean HOMA-IR level is significantly lower among LRYGB patients than LSG patients (1.18 vs 2.71, p = 0.0066).
3. Two-week mean PBM *ND1/18S rRNA* level is significantly lower among LRYGB patients than LSG patients (0.52 vs 0.96, p = 0.0025).

No other comparisons resulted in significant differences, probably due to small sample sizes. Some had only one patient in one group.

Supplemental Table S4. Comparison of *ND6* and PBM *ND1/18SrRNA* in HC and Obese, BS by factor: MetS, T2D or none.

	T2D/MetS/none	N	Mean	Std Dev	Comparison with HC (Dunnett's method)
Plasma *ND6*	HC	7	0.01	0.01	
	MetS	4	0.04	0.04	0.2233
	none	2	0.04	0.01	0.5677
	T2D	5	0.07	0.03	0.0098
PBM *ND1/18S rRNA*	HC	8	0.76	0.32	
	MetS	4	0.96	0.28	0.6185
	none	2	1.29	0.25	0.1228
	T2D	7	0.94	0.33	0.5570

Supplemental Table S5. Comparison of *ND6* and PBM *ND1/18SrRNA* in HC and Obese, BS group where Obese group is classified as IR or none.

	IR/None	N	Mean	Std Dev	Comparison with HC (Dunnett's method)
Plasma *ND6*	HC	7	0.01	0.01	
	IR	9	0.05	0.03	0.0119
	none	2	0.04	0.01	0.4599
PBM *ND1/18S rRNA*	HC	8	0.76	0.32	
	IR	11	0.95	0.30	0.3200
	none	2	1.29	0.25	0.0759

Supplemental Table S6. Comparison of plasma *ND6* in Obese group by MetS, T2D, or none

		Plasma *ND6*					P (ANOVA)
**Time**	**T2D/Mets/none**	**N**	**Mean**	**Std Dev**	**Min**	**Max**	
BS	Mets	2	0.052	0.036	0.0264	0.0769	0.7613
	none	0					
	T2D	4	0.061	0.032	0.0234	0.1004	
2W	Mets	2	0.055	0.045	0.0231	0.0867	0.7793
	none	2	0.043	0.061	0.0001	0.0858	
	T2D	5	0.087	0.092	0.0061	0.1912	
3M	Mets	2	0.143	0.057	0.1031	0.1830	0.1645
	none	1	0.005		0.0049	0.0049	
	T2D	4	0.062	0.048	0.0001	0.1174	
6M	Mets	1	0.044		0.0441	0.0441	0.4869
	none	2	0.014	0.020	0.0004	0.0282	
	T2D	5	0.100	0.092	0.0260	0.2601	

No significant differences are observed. Note that sample sizes are very small.

Supplemental Figure Legends:

Figure S1. Correlation between HgbA1c and plasma *ND6* levels prior to surgery with patients separated as HC and Obese, BS (n=7, HC; n=11, Obese, BS).

Figure S2. Effect of bariatric surgery on plasma cf-mtDNA. *ND6* level in obese patients prior to surgery and at the indicated time points follow up post-surgery. Data are presented as boxplots with mean marked by green lines, n = 6-9.

Figure S3. Schematic of location of primers and long overlapping mtDNA sequences (Mito1 and Mito2) amplified whole mitochondrial genome during long-range PCR.

Figure S4. Effect of obesity, MetS and T2D on the mtDNA abundance in PBM cells. Data are presented as boxplots with mean marked by green lines. **A**- mtDNA content in PBM cells from Obese patients and HC (n=8-13). (**B-D**) mtDNA abundance in PBM cells demonstrated by group **B-** data in Obese, BS group were plotted according to the MetS, T2D or none (n = 2-8); **C**- data in Obese, BS group were plotted according to the IR or none (no IR); n = 2-8 subjects per group; **D**- data in Obese, BS group were plotted according to the T2D or non-T2D (n = 6-8).

Supplemental Figures

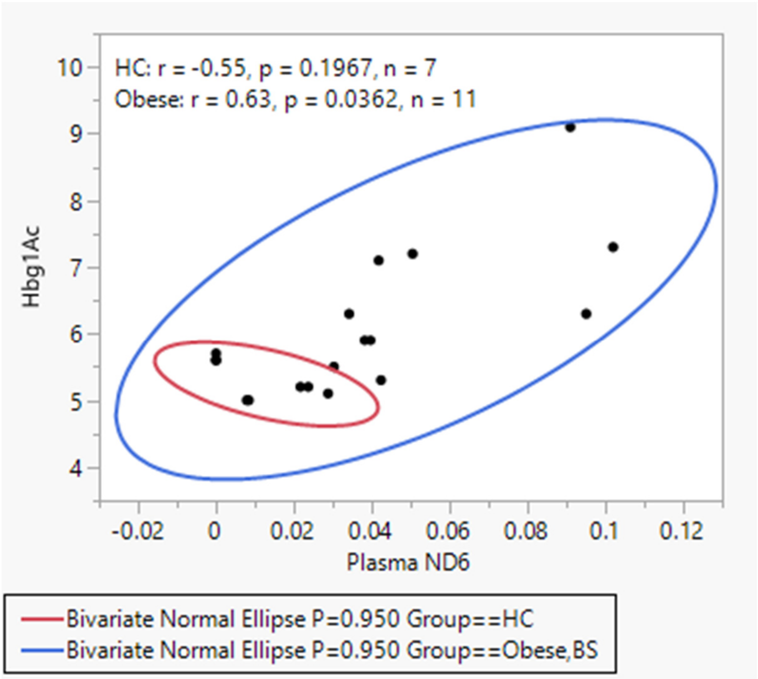

Figure S1. Correlation between HgbA1c and plasma *ND6* levels prior to surgery with patients separated as HC and Obese, BS (n=7, HC; n=11, Obese, BS).

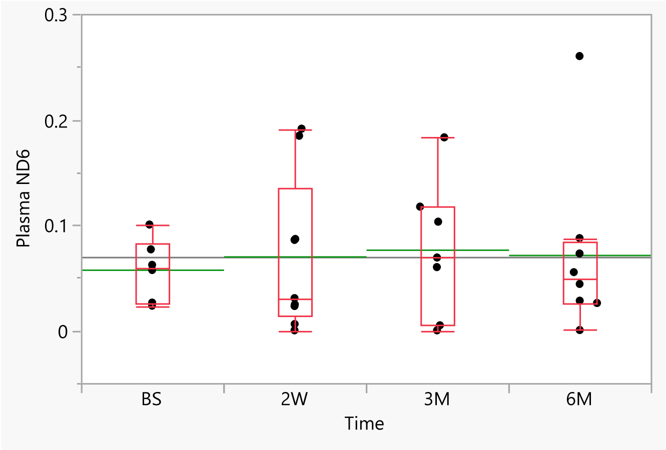

Figure S2. Effect of bariatric surgery on plasma cf-mtDNA *ND6* level in obese patients prior to surgery and at the indicated time points follow up post-surgery. Data are presented as boxplots with mean marked by green lines, n = 6-9.

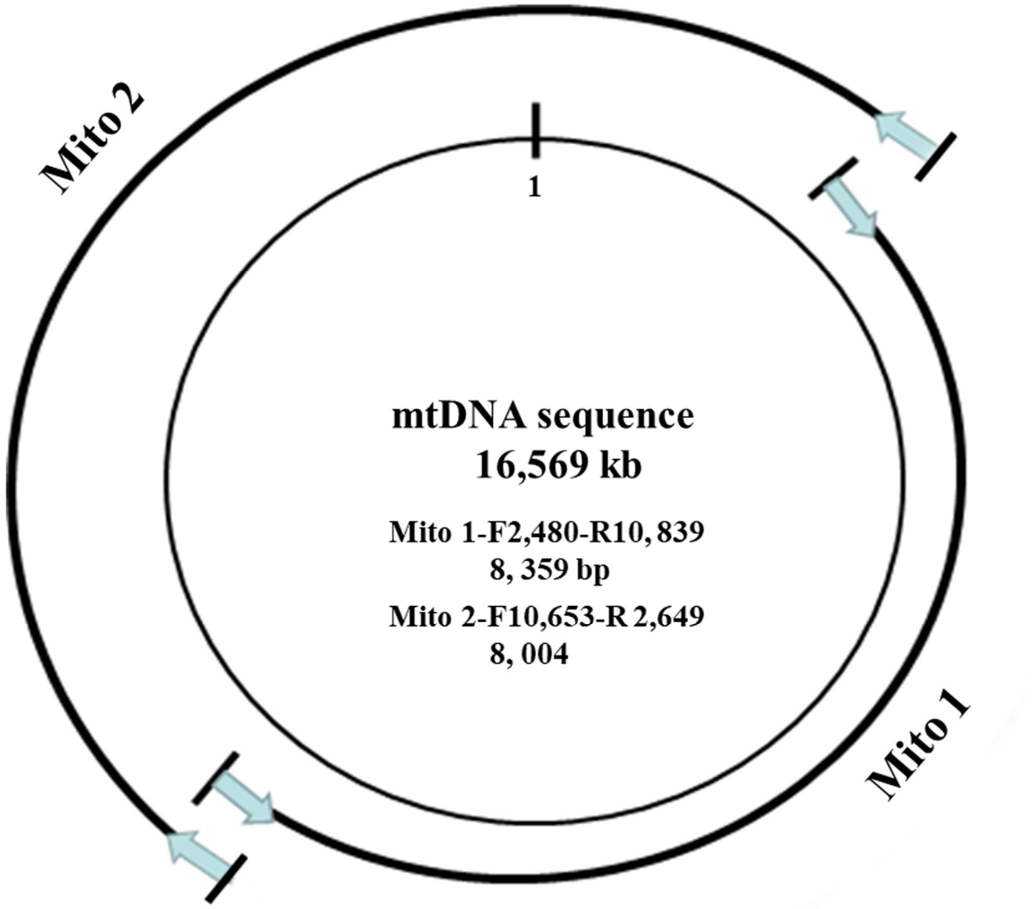

Figure S3. Schematic of location of primers and long overlapping mtDNA sequences (Mito1 and Mito2) amplified whole mitochondrial genome during long-range PCR.

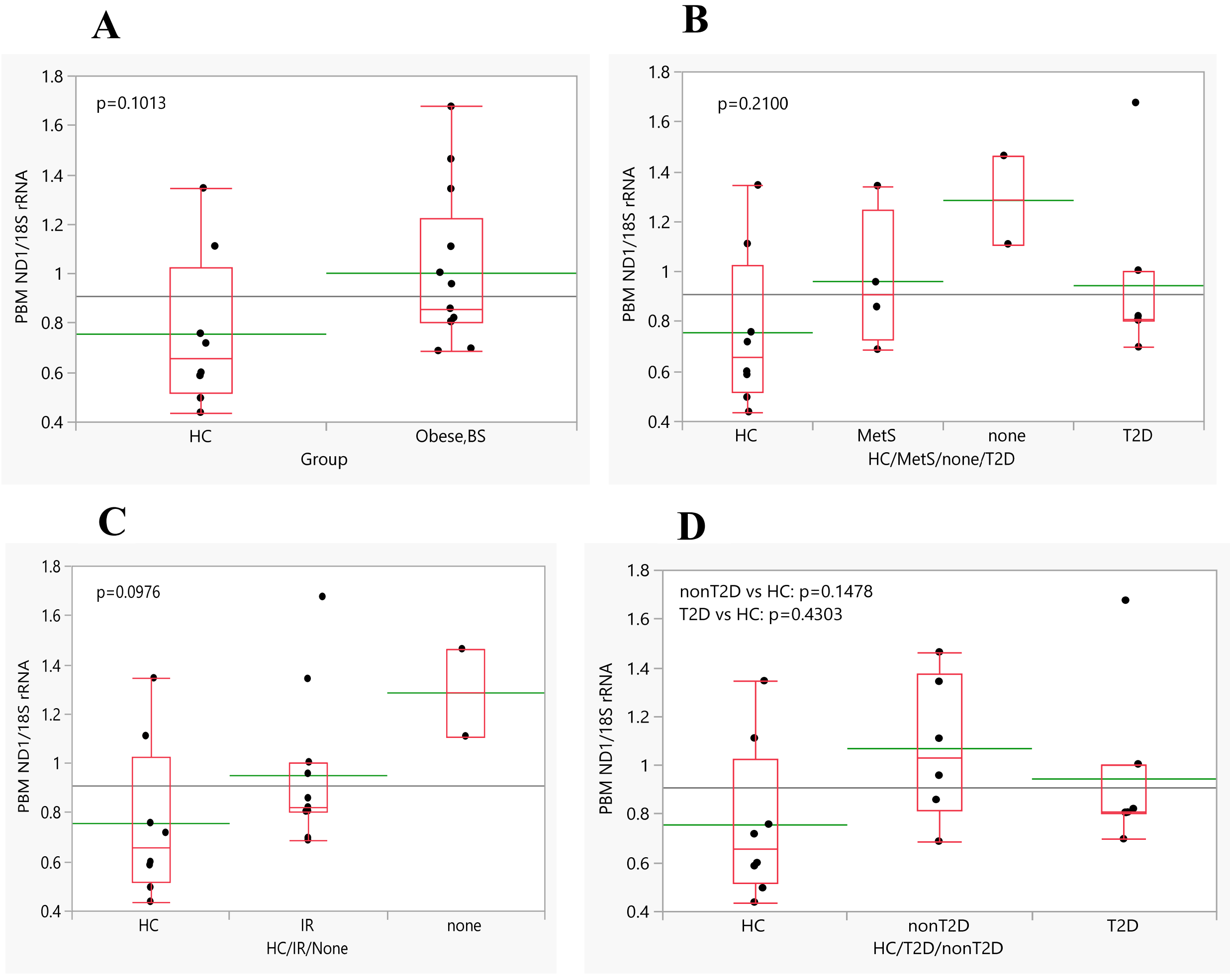

Figure S4. Effect of obesity, MetS and T2D on the mtDNA abundance in PBM cells. Data are presented as boxplots with mean marked by green lines. **A**- mtDNA content in PBM cells from Obese patients and HC (n=8-13). (**B-D**) mtDNA abundance in PBM cells demonstrated by group **B-** data in Obese, BS group were plotted according to the MetS, T2D or none (n = 2-8); **C**- data in Obese, BS group were plotted according to the IR or none (no IR); n = 2-8 subjects per group; **D**- data in Obese, BS group were plotted according to the T2D or non-T2D (n = 6-8).
